# Indoor Air Quality Conditions and Respiratory Virus Detections in Elementary Schools—Kansas City, Missouri, February–March 2023

**DOI:** 10.1155/ina/9935344

**Published:** 2025

**Authors:** Olivia M. Almendares, Jasmine D. Ruffin, Luke C. Gard, Rangaraj Selvarangan, Anjana Sasidharan, Dithi Banerjee, Nibha Sagar, Amanda Hayes, Sydnie Petty, Brian R. Lee, Christopher Young, Janelle Porter, Shannon Tilsworth, Jennifer L. Goldman, Jennifer E. Schuster, Hannah L. Kirking

**Affiliations:** 1 National Center for Immunization and Respiratory Diseases, Centers for Disease Control and Prevention, Atlanta, Georgia, USA; 2 Eagle Health Analytics, Peachtree Corners, Georgia, USA; 3 Children’s Mercy Kansas City, Kansas City, Missouri, USA; 4 North Kansas City School District, Kansas City, Missouri, USA

## Abstract

**Background/Objectives:**

Respiratory viruses circulate year-round and can spread indoors via inhalation of airborne particles. Effective ventilation and filtration may reduce transmission, particularly in school settings where children and staff spend significant time. This study examines the impact of indoor air quality (IAQ) and ventilation in schools on respiratory virus detection.

**Methods:**

During February 27 to March 30, 2023, we assessed the relationship between IAQ and respiratory viruses in two Kansas City, Missouri, elementary schools by collecting bioaerosol samples, student and staff nasal swabs, and monitoring IAQ across 10 rooms (six classrooms, two common spaces, and two nurses’ offices). We calculated air changes per hour (ACH) and analyzed viral detections in nasal swabs and bioaerosol samples by high (≥ 1000 ppm) or low (< 1000 ppm) CO_2_ concentration in classrooms.

**Results:**

We collected 173 nasal swabs from 100 participants and 30 bioaerosol samples from 10 rooms. Participants were mostly female (68%) and white (60%). Viruses were detected in 90% of bioaerosol and 35% of nasal samples. Four classrooms and both common spaces had the same virus detected in bioaerosol and nasal swab samples. None of the spaces met the CDC recommended ACH of 5, and only one exceeded ANSI/ASHRAE 62.1-2022 standards for outdoor air supply. During school hours, 83% of classrooms had high average CO_2_ concentrations. Classrooms with high CO_2_ levels had higher viral detection.

**Conclusions:**

Viral presence was found in bioaerosol and nasal samples with some overlap in concurrently collected samples. Findings suggest a possible connection between high CO_2_ levels and virus detection. Improving IAQ and ventilation may reduce respiratory infection risks in schools. However, this study highlights the need to further assess the impact of various environmental modifications on respiratory virus transmission in schools, including determining optimal strategies such as ventilation, filtration, or germicidal ultraviolet energy.

## 1. Introduction

Respiratory viruses circulate year-round, following predictable seasonal patterns. Person-to-person transmission occurs via several routes: inhalation of aerosolized particles (i.e., airborne—long or short range), respiratory sprays from coughs or sneezes landing in mucus membranes (e.g., eyes and nostrils), and contact transmission via direct (i.e., handshake) or indirect means (i.e., contaminated surfaces) whereby infectious particles are transferred to mucus membranes [1, 2]. Many infections are acquired indoors, through inhalation of viral airborne particles; however, this can vary depending on the specific virus and indoor environmental conditions. This underscores the potential importance of adequate ventilation and filtration to reduce transmission risk [3, 4]. Schools are critical environments for respiratory virus prevention. Children spend substantial time there and may be more vulnerable to poor indoor air quality (IAQ) as children have higher breathing rates than adults, their lung function may still be evolving (e.g., reactive airway disease or chronic lung disease from prematurity), and their immune systems are still developing [5, 6]. Limited evidence suggests high respiratory virus transmission in schools [7], highlighting the need for effective prevention strategies.

Bioaerosol sampling, coupled with IAQ assessment, provides a simpler, less costly alternative to human sample collection, which can be labor-intensive and dependent on participation [8, 9]. Improved IAQ and ventilation have been associated with reduced illness-related student absences, including respiratory illnesses [10, 11]. Although data directly linking school ventilation with laboratory-confirmed infections are limited, one study reported a higher incidence of acute respiratory illness (ARI) in college dormitories with low outdoor air (OA) supply rates [12]. During the COVID-19 pandemic, school-based surveillance programs used routine testing to maintain in-person learning [13, 14] and innovative approaches such as wastewater and bioaerosol sampling gained attention for monitoring respiratory viruses [8, 15, 16]. Notably, bioaerosol and nasal swab data have shown consistent trends in severe acute respiratory syndrome coronavirus 2 (SARS-CoV-2) and influenza activity in schools [9].

In this context, heating, ventilation, and air conditioning (HVAC) systems in US buildings play a crucial role in minimizing airborne pathogen transmission. The Centers for Disease Control and Prevention (CDC) recommends a minimum ventilation rate of five air changes per hour (ACH). While not a hard-and-fast rule, this target serves as a general benchmark for air exchange likely to help reduce viral particles [2, 17]. Most HVAC systems follow American National Standards Institute and the American Society of Heating, Refrigerating and Air-Conditioning Engineers (ANSI/ASHRAE) standards, which historically have not addressed infectious aerosol control. The recent ASHRAE standard 241 introduces infection risk management mode (IRMM) to control indoor infectious aerosols during high-risk periods, though implementation criteria remain undefined. Moreover, ASHRAE guidelines are voluntary and may not be adopted unless incorporated into building codes. Upgrading school HVAC systems to meet current standards is often constrained by building design and costs, making widespread implementation difficult even with established guidelines. A longstanding gap remains between engineering and epidemiologic approaches: Engineers prioritize built environment characteristics, IAQ indicators, and modeling, while epidemiologists focus on health outcomes. This gap has hindered integrated understanding of indoor transmission. A pre-COVID-19 pandemic systematic review highlighted the need for studies integrating building and ventilation characteristics, airborne exposures, and human disease outcomes [18].

To address this gap, we conducted a pilot study in two elementary schools participating in a school-based respiratory virus surveillance program [19]. We collected nasal swabs, bioaerosol samples, and IAQ data to explore associations between classroom environmental conditions and respiratory viral detections. This report describes ventilation rates and IAQ indicators (i.e., CO_2_, relative humidity (RH), and temperature), compares virus detection across sampling methods, and evaluates CO_2_ levels as a proxy for adequate ventilation in classrooms.

## 2. Materials and Methods

### 2.1. Study Design and Population.

During February 27–March 30, 2023, we collected data from two elementary schools (School A and School B) in Kansas City, Missouri. We studied six classrooms (three per school), two common spaces (a cafeteria and a multipurpose room also used as a cafeteria), and two nurses’ offices (one per school), totaling 10 rooms. We collected bioaerosol samples, human nasal swabs, and conducted IAQ monitoring. We also recorded building and environmental data including room dimensions, supply and return airflows, occupancy, and natural or mechanical ventilation factors (e.g., open/closed doors and windows).

**Figure 1:**
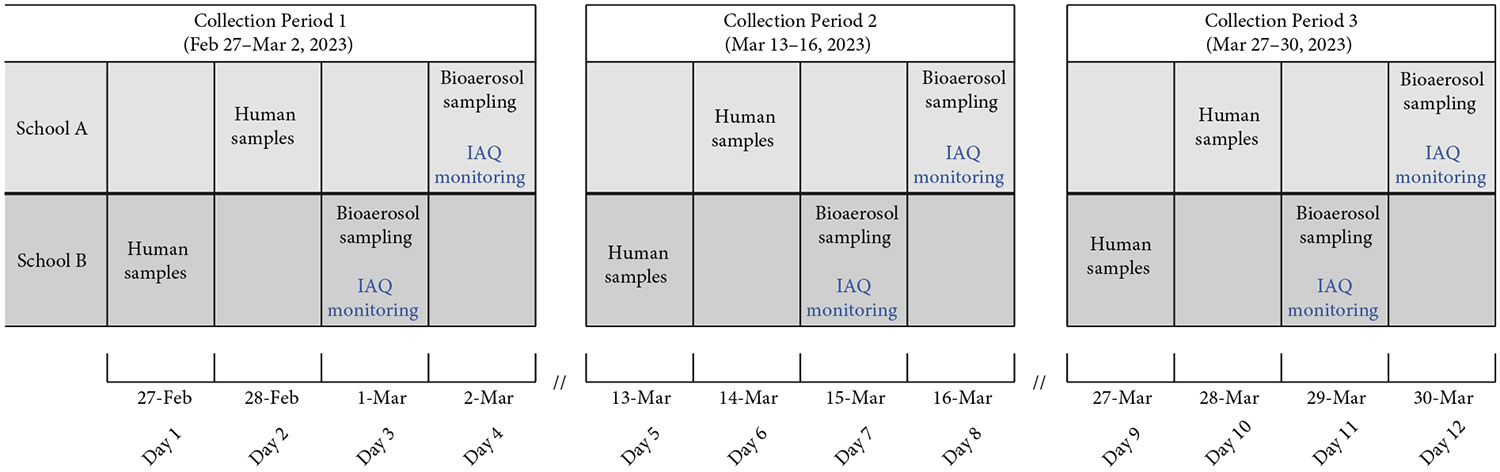
Timeline of bioaerosol, respiratory samples, and indoor air quality indicator collection in two elementary schools. Nasal swabs were collected from student and staff participants both as part of monthly surveillance and as on-demand symptomatic samples, which could be submitted at any point during the collection periods. Bioaerosol samples were collected using the AerosolSense sampler during school hours on designated days. Indoor air quality indicators—CO_2_, temperature, RH, and Particulate Matter (2.5 *μ*m and 10 *μ*m)—were measured using the AirAssure 8144-6 Indoor Air Quality Monitor during school hours on the indicated days. Note: IAQ, indoor air quality.

Data collection took place over three, 4-day periods (12 days total): February 27–March 2 (Collection Period 1), March 13–16 (Collection Period 2), and March 27–30 (Collection Period 3). During the 2022–2023 school year, all students in prekindergarten through fifth grade and school staff were eligible to voluntarily enroll in a respiratory virus surveillance program. Participants provided self-collected nasal swabs 48 h before each bioaerosol and IAQ sampling period (Figure 1).

### 2.2. Student and Staff Data and Nasal Swab Collection.

Nasal swabs were self-collected monthly at school under the supervision of study staff or the school nurse, regardless of symptoms (surveillance samples). Additionally, on-demand nasal swabs were collected from participants experiencing ARI (e.g., cough, runny nose, and fever) either at home or school (“on-demand” symptomatic samples). All participants and/or parents/legal guardians of participants provided electronic consent to participate and detailed methods of School KIDS surveillance are previously described [19]. For this ventilation-focused substudy, only nasal swabs collected during the three, 4-day periods were included in the analysis.

Demographic information, including age, gender, household members, student enrollment in the National School Lunch Program (for free or reduced-price lunch), and influenza and COVID-19 vaccination status, was collected via parental and staff surveys at the time of enrollment. Vaccination status was further verified through state immunization registries and medical records. Race and ethnicity were categorized as White, non-Hispanic/Latino (NH); Hispanic/Latino; and some other race, NH, which combined groups individually comprising < 10% of the population. This included Black, NH; Asian, NH; Native Hawaiian or Pacific Islander, NH; and multiracial, NH.

### 2.3. Room, Building, and Occupancy Data.

All evaluated spaces were in fixed, enclosed buildings constructed in the mid to late 1950s. Both schools were equipped with HVAC systems using pleated MERV8 filtration and controlled by simple on/off thermostats. We collected room dimensions (length, width, and height in feet) for all spaces where bioaerosol sampling was conducted and calculated room area and volume. Expected occupancy was based on school classroom occupancy load standards which account for the amount of space per student (e.g., rooms 750–1000 ft^2^ were assumed to have an expected occupancy up to 25 people) [20, 21]. No spaces in either school incorporated the use of additional air cleaning devices such as portable air cleaners (PACs) or air treatment such as upper-room germicidal ultraviolet (GUV) fixtures.

Observed room occupancy, natural ventilation (open windows and doors), and mechanical ventilation (HVAC on/off) were recorded every 30 min during school hours for 1 day per collection period coinciding with bioaerosol sample collection. These ventilation conditions were used as a proxy for the entire collection period. HVAC run-time was estimated by averaging the system’s operational time across observed days. Average occupancy was calculated using observed occupancy data, including the average of the maximum number of occupants across all collection periods, and the observed occupancy at 50% of this average.

### 2.4. Ventilation and ACH.

We defined an air change rate (or ACH) as the number of times the equivalent volume of room air is replaced by air that is free of viral particles over an hour. This includes the amount of OA introduced to the space and any air cleaning (i.e., capture of viral particles via use of filters in HVAC system or PACs) or air treatment (i.e., inactivation of viral particles through use of GUV energy). Air cleaning and air treatment systems can provide equivalent ACH yet are not a substitute for ensuring code-compliant minimum OA delivery. In the following, we outline the steps taken to calculate the final clean ACH.

Supply and return airflow velocities were measured (in feet per minute) from all registers in each room using the VelociCalc Air Velocity Meter 9545A [22]. The diameter for each duct was programed into the instrument, and for each supply register, airflow measurements were taken at six evenly spaced points across the vent area. For return vents, airflow measurements were taken at four evenly spaced points. Measurements were averaged to calculate the total measured supply airflow rate (in cubic feet per minute). This total was then normalized based on the estimated HVAC system run-time for each evaluated space as

$$\text{Normalized Supply Air Rate}\;{\left(\text{NSAR}\right)}_{\text{cfm}}\;\;=({\text{Total measured supply air}}_{\text{cfm}})\;\;\times (\%\text{HV AC system runtime}).$$

To estimate the recommended OA rate for each space, we referenced Table 6-1 from ANSI/ASHRAE Standard 62.1-2022 [23], which provides minimum recommended OA rates for occupiable indoor spaces, expressed as air flow per person (in cubic feet per minute /person or $R_{p}$) and air flow per area (in cubic feet per minute per square foot of area or $R_{a}$). Using the occupancy category (including classrooms: ages 5–8; classrooms: ages 9 plus; daycare sickroom; and multiuse assembly), the number of observed occupants, and the room’s floor area, we calculated the recommended amount of OA for each evaluated space as follows:

$${\text{OA}}_{\text{cfm}},\text{recommended}=(R_{p}\times \text{number of observed occupants})\;\;+(R_{a}\times {\text{Room area in ft}}^{2}).$$

We assumed the outdoor intake damper was open at 10%, consistent with typical settings for facilities in this region, balancing fresh air intake with temperature and RH variations. The estimated amount of OA supplied to the space was calculated as ${\text{OA}}_{\text{cfm},}\text{estimated}=({\text{NSAR}}_{\text{cfm}})\times 0.10$. All supply and OA rates calculated in cubic feet per minute for each space were also converted to metric units: liters per second.

Using these OA estimates, we calculated air changes per hour for OA (${\text{ACH}}_{\text{OA}}$) as follows:

$${\text{ACH}}_{\text{OA}}(\text{recommended or estimated})\;\;=\frac{{\text{OA}}_{\text{cfm}}[\text{recommended or estimated}]\times 60}{{\text{Room volume in ft}}^{3}}.$$

For return air, we estimated that 90% of the NSAR to the space represents return or recirculated air (RA). As noted in Table 12-1 of ANSI/ASHRAE Standard 52.2-2017 [23], MERV8 filters are 20% efficient at filtering particles between 1.0 and 3.0 *μ*m. Thus, 20% of RA was considered infectious-free air [23]. The formula for return air ACH (${\text{ACH}}_{\text{RA}}$) is as follows:

$${\text{ACH}}_{\text{RA}}=\frac{(({\text{NSAR}}_{\text{cfm}}\times 0.90)\times 0.2)\times 60}{{\text{Room volume in ft}}^{3}}.$$

Finally, we calculated the total clean ACH for each space by summing ${\text{ACH}}_{\text{OA}}$ and ${\text{ACH}}_{\text{RA}}$ [24].

### 2.5. Bioaerosol Sample Collection.

All evaluated spaces were sampled for respiratory viruses using the AerosolSense sampler, which operates at an airflow rate of 7.06 cfm (200 L/min). Sampling was conducted for an average of nine continuous hours per collection period, corresponding to the typical classroom hours. A single AerosolSense cartridge was used for each air sample collected. Bioaerosol samplers were placed along interior walls at least 2 ft. above the floor and on average 15–20 ft. from hallway doors to minimize influence of hallway conditions. Samplers were also positioned near return vents in both schools to capture air representative of room conditions. The proportion of air volume sampled, based on the static room volume, was calculated as Air Volume Sampled = Sampling time in minutes × Sampling rate of 7 06 cfm.

### 2.6. IAQ Indicators and Air Stuffiness Index.

In each evaluated space, we used the AirAssure 8144-4 IAQ Monitor to measure IAQ indicators including CO_2_ (in parts per million), particulate matter (PM_2.5_, PM_10_ in micrograms per cubic meter), temperature (in Fahrenheit), and RH (in percent). Temperature was also converted to metric units as degrees Celsius. Monitors were calibrated in late February, prior to the start of data collection. Instruments were placed in the room as close to the middle of the room as possible and away from windows, doors, and out of the direct path of supply air vents. The instrument recorded data every 60 s for approximately nine continuous hours per collection period, including an hour before and after school hours. For analysis, we calculated the 7-h mean for all IAQ indicators to assess IAQ during occupied hours, excluding pre and postschool hours. No missing sensor data were noted when restricting to occupied school hours. The nondispersive infrared sensors had a resolution of 1 ppm, with an accuracy of the larger of 3% or 30 ppm within the range of 400–10,000 ppm.

An air stuffiness or ICONE *(Indice de CONFinement d’air dans les Ecoles)* index [25] was developed in 2011 by French researchers as a communication tool to evaluate air stuffiness during occupied periods. It uses CO_2_ thresholds of 1000 and 1700 ppm, classifying air quality into levels: $n_{0}$ —values ≤ 1000 ppm, $n_{1}$ —values between 1000 and 1700 ppm, and $n_{2}$ —values > 1700 ppm. CO_2_ concentrations measured during normal classroom attendance were used to calculate the ICONE index. Normal classroom attendance was defined as at least half of the observed number of students present for a minimum of 5 h during the school day. The index was calculated by applying the equation:

$$\text{ICONE}=\left(\frac{2.5}{\log_{10}(2)}\right)\log_{10}(1+f_{1}+3f_{2}),$$

where $f_{1}$ is the proportion of CO_2_ values between 1000 and 1700 ppm ($f_{1}=n_{1}∕(n_{0}+n_{1}+n_{2})$) and $f_{2}$ is the proportion of CO_2_values > 1700 ppm ($f_{2}=n_{2}∕(n_{0}+n_{1}+n_{2})$). Results are rounded to the nearest whole number, yielding a score from 0 to 5. A score of 0 indicates nonstuffy air (CO2 concentration < 1000 ppm 100% of the time), while a score of 5 indicates extremely stuffy air (CO2 concentration > 1700 ppm 100% of the time). Scores could not be calculated for the cafeteria or multipurpose room due to insufficient occupancy.

### 2.7. Laboratory Viral Testing.

Bioaerosol samples and human nasal swabs were tested for 15 common respiratory viruses (adenovirus [AdV], human seasonal coronaviruses [229E, HKU1, NL63, OC43] [HCoV], human metapneumovirus [hMPV], influenza [FluA, FluB], parainfluenza virus Types 1, 2, 3, 4 [PIV], respiratory syncytial virus [RSV], rhinovirus/enterovirus [RV/EV], and SARS-CoV-2) by multiplex real-time polymerase chain reaction (PCR). Bioaerosol and nasal swab surveillance samples were tested using Panther Fusion assays (Hologic Inc., Marlborough, Massachusetts) while nasal swab “on-demand” symptomatic samples were tested using the QIAstat-Dx assay cartridge (QIAGEN, Germantown, Maryland). Molecular typing of all bioaerosol and nasal swab samples positive for AdV and RV/EV was conducted using PCR amplification and sequencing of the partial hexon gene and VP4/VP2 capsid gene region, respectively [26, 27]. For RV/EV typing, targeting the VP4/VP2 capsid gene region allows for typing of most rhinoviruses and identification of select enterovirus types. AdV or RV/EV detections were defined as nontypeable when the sample was unable to be sequenced (but typing was attempted).

### 2.8. Statistical Analyses.

Demographics, vaccination status (Influenza and COVID-19), and nasal swab characteristics for participating students and staff were described. Environmental data, including building and room characteristics and variability of IAQ measures, were also described. Ventilation rates for all evaluated spaces were calculated, and we compared the recommended and estimated OA rates to assess whether ventilation in each evaluated space met the ANSI/ASHRAE 62.1-2022 standard.

The proportion of bioaerosol and nasal swab samples with detection of at least one virus or codetection of two or more respiratory viruses was evaluated. Concordance of viral detections between bioaerosol and nasal swab samples was assessed by room type and collection period. Concordance was defined as the simultaneous detection of the same virus in both sample types, meaning at least one virus detected from a nasal swab had to match the virus detected in the corresponding bioaerosol sample, within the same room and collection period. For classrooms, this involved comparing bioaerosol samples with nasal swabs from staff and students assigned to that room and collected during the same period (48h prior to bioaerosol sampling). For the cafeteria and multipurpose room, nasal swabs from all student participants, including those from classrooms without bioaerosol sampling, were compared with bioaerosol samples from these rooms, as teachers do not eat lunch there.

CO_2_ concentrations were classified as high (≥ 1000 ppm) or low (< 1000 ppm) based on the 7-h mean for each collection period and classroom. We then determined the proportion of respiratory viral detections in nasal swabs and bioaerosol samples by high and low CO_2_ concentrations. Statistical associations were assessed using chi-square for comparisons between CO_2_ levels and viral detections in nasal swabs and Fisher’s exact test for associations between CO_2_ levels and viral detections in bioaerosol samples. All analyses were conducted using SAS software (Version 9.4; SAS Institute).

This activity was reviewed by the research ethics boards at both Children’s Mercy Kansas City (CMKC) and CDC, deemed not research, and was conducted in accordance with applicable federal law and CDC policy [28].

## 3. Results

### 3.1. Enrollee Demographics, Vaccination Status, and Nasal Swab Characteristics.

During the 12-day period encompassing all three collection periods, 173 nasal swabs were collected from 100 participants—61% students (median age: 8.4 years; interquartile range [IQR]: 6.8–10.2) and 39% staff (median age: 39.6 years; IQR: 31.5–53.5). School A contributed 62% of all participants (40 students and 22 staff), while School B comprised 38% (21 students and 17 staff). The majority of participants were female (68% overall; 56% students and 87% staff) and identified as white, NH (60%); a little over one-third (34%) came from households with five or more members. Overall, 16% of participants received the 2022-2023 COVID-19 bivalent vaccine and 36% received the 2022-2023 seasonal influenza vaccine.

Of the 61 students participating in nasal swab collection and testing across both schools, 17 (28%) were from classrooms where bioaerosol sampling was conducted. Similarly, 8 (21%) of the 39 staff participants were from these classrooms. Of the total 173 nasal swabs collected, 166 (96%) were surveillance swabs, and 7 (4%) were requested by participants as on-demand swabs due to experiencing ARI symptoms, although participants could have had ARI symptoms at the time of monthly surveillance swab collection (Table 1).

### 3.2. Room Occupancy.

During all collection periods, the observed average classroom occupancy was similar between schools: 22 occupants (range: 18–40) for School A and 19 (range: 16–30) for School B. The nurses’ offices also had similar average occupancy to each other. For common spaces, School A’s cafeteria averaged 78 (range: 65–105) occupants, while School B’s multipurpose room averaged 93 (range: 80–105).

During school hours, classrooms in School A were at 50% of their observed average occupancy 71% of the time, compared to 61% in School B. Nurses’ offices were at 50% occupancy 76% of the time in School A and 73% in School B. During lunch hours, School A’s cafeteria and School B’s multipurpose room reached 50% occupancy for 50% and 60% of the time, respectively (Table 2).

### 3.3. Ventilation.

In School A classrooms, internal doors were open 74% of the time and HVAC run-time was 24% compared with School B classrooms at 59% and 5%, respectively. School A’s cafeteria had doors (internal to hallway) open 100% of the time and HVAC running 76% of the time, while School B’s multipurpose room had open doors 51% of the time and HVAC running 47%. Windows remained closed in all observed spaces with existing or operable windows (Table 2).

After normalizing for HVAC run-time, the NSAR was 215 cfm (101 L/s) for School A classrooms and 373 cfm (176 L/s) for School B classrooms. The recommended OA rate was 321 cfm (151 L/s) for School A classrooms and 295 cfm (139 L/s) for School B. However, estimated OA rates were much lower: 21 cfm (10 L/s) for School A and 37 cfm (17 L/s) for School B classrooms; thus, no classroom met the ANSI/ASHRAE 62.1-2022 standard for OA supply rate. Only School A’s cafeteria exceeded the recommended OA rate, with an estimated rate of 794 cfm (375 L/s) versus the recommended 761 cfm (359 L/s) (Table 2).

**Figure 2:**
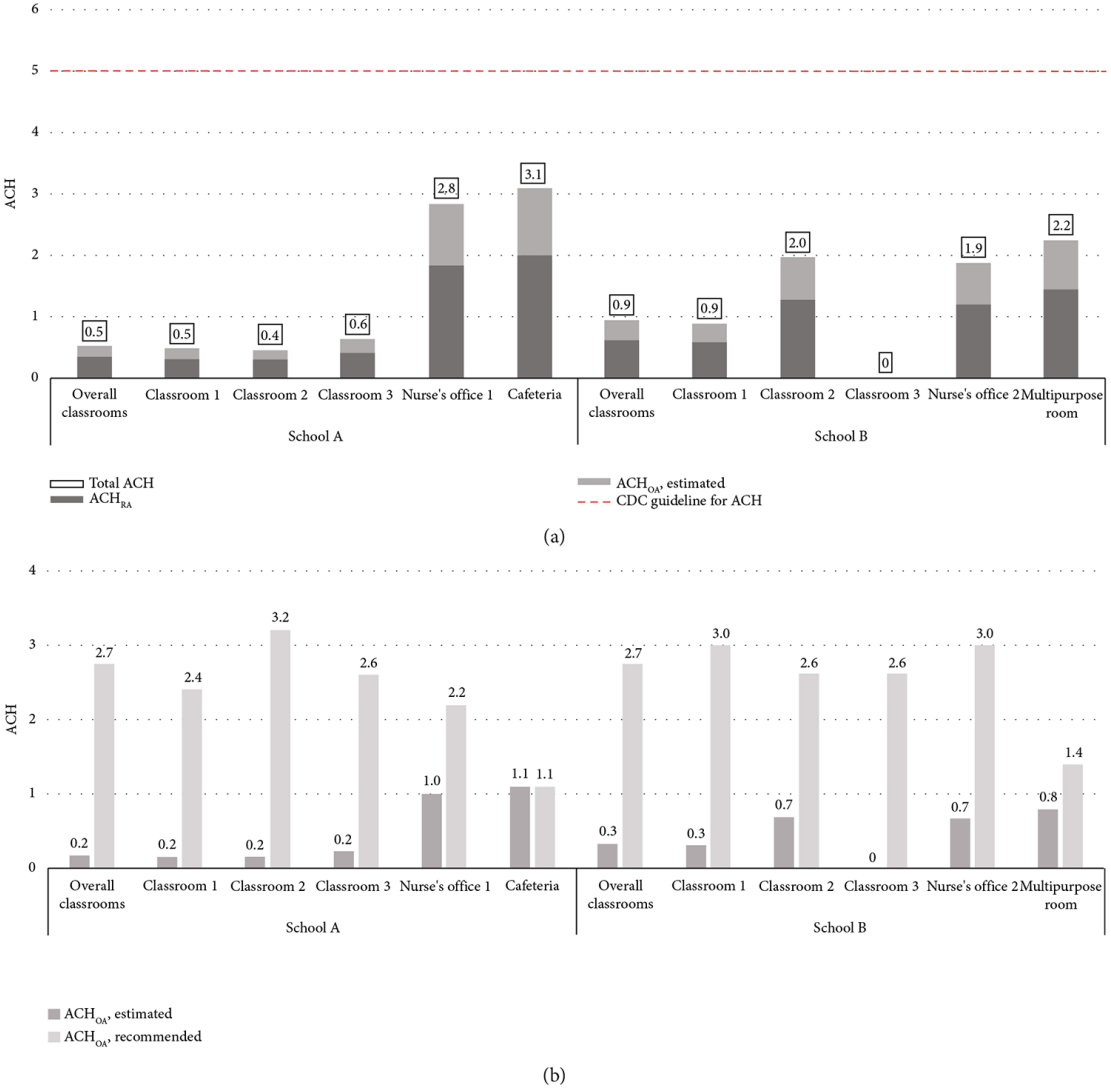
Ventilation rates in two schools, February–March 2023 comparing (a) total ACH, ${\text{ACH}}_{\text{RA}}$, and estimated ${\text{ACH}}_{\text{OA}}$ versus CDC ACH guidelines and (b) estimated ${\text{ACH}}_{\text{OA}}$ versus recommended outdoor air levels by room type based on ASHRAE 62.1-2022 standards. Total ACH reflects the sum of ${\text{ACH}}_{\text{OA}}$ (outdoor air) and ${\text{ACH}}_{\text{RA}}$ (filtered return air). ${\text{ACH}}_{\text{RA}}$ represents 90% of the normalized supply air, adjusted by a 20% MERV8 filter credit, converted to hours based on room volume. Estimated ${\text{ACH}}_{\text{OA}}$ accounts for 10% of the normalized supply air, converted to hours based on room volume. Recommended ${\text{ACH}}_{\text{OA}}$ is based on ASHRAE 62.1-2022 outdoor air standards, also converted to hours based on room volume. In School B’s Classroom 3, the HVAC system was off during observed periods, so ACH could not be estimated. Note: ACH, air changes per hour; RA, return air; OA, outdoor air.

The estimated ${\text{ACH}}_{\text{OA}}$ for classrooms were lower than recommended: 0.2 versus 2.7 for School A and 0.3 versus 2.7 for School B. Only School A’s cafeteria met OA recommendations at 1.1 (Figure 2b).

The estimated total clean ACH, which accounts for both outdoor and filtered ACH (i.e., ${\text{ACH}}_{\text{OA}}$ and ${\text{ACH}}_{\text{RA}}$, respectively), was low for classrooms: 0.5 for School A and 0.9 for School B. Despite higher estimated ${\text{ACH}}_{\text{OA}}$ for School A’s cafeteria, which met code for OA, total clean ACH values remained low: 3.1 for School A cafeteria and 2.2 for School B’s multipurpose room. All evaluated spaces had total clean ACH values well below the CDC’s recommended ACH of 5 or higher (Figure 2a).

### 3.4. IAQ Conditions.

**Figure 3:**
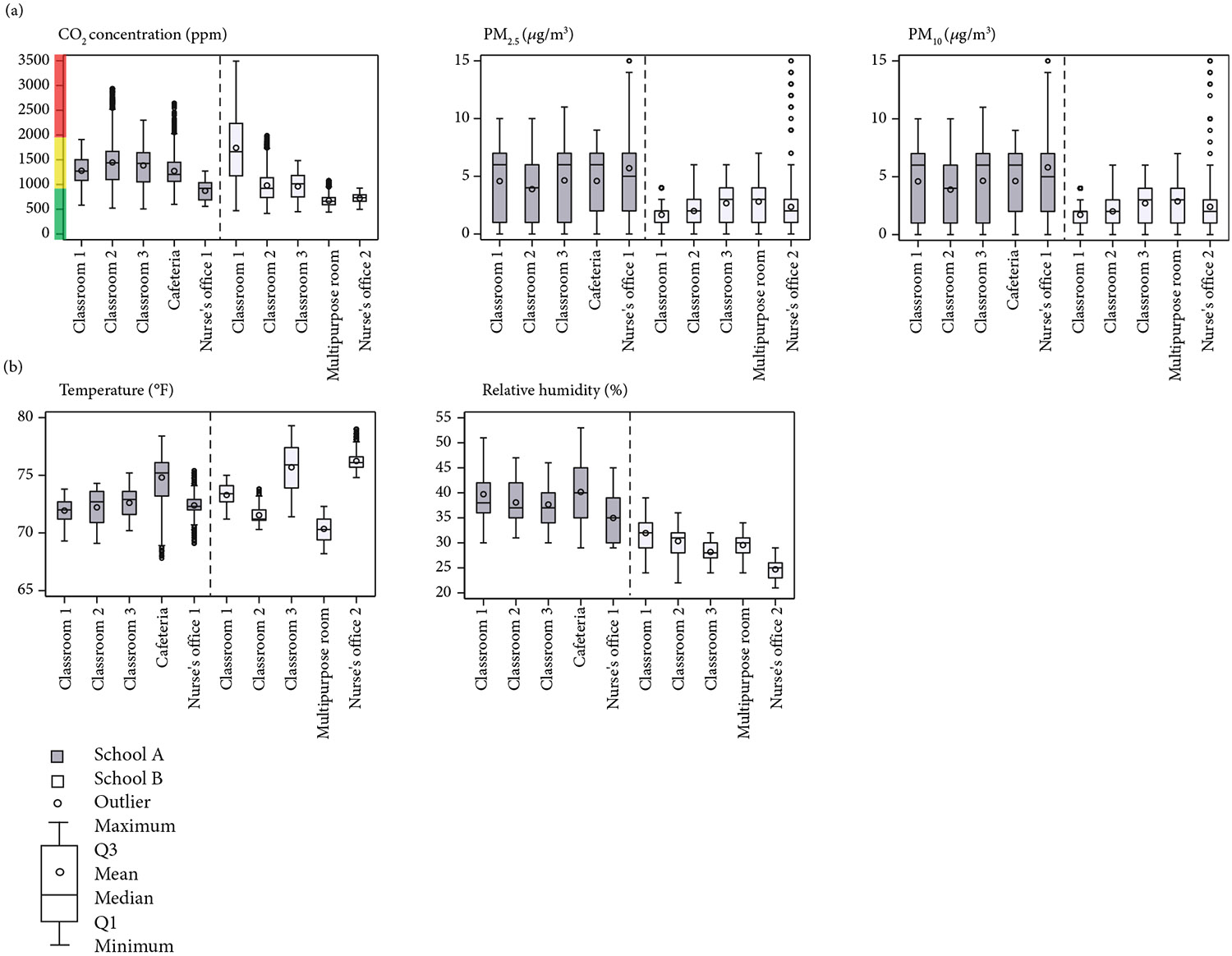
Indoor air quality (IAQ) variability by room type in two elementary schools—Kansas City, Missouri, February–March 2023 as (a) CO_2_, PM_2.5_, and PM_10_ and (b) temperature and relative humidity. IAQ indicators were measured during school hours using the TSI AirAssure 8144–6 monitor. Mean hourly values were calculated across all collection periods during occupied hours. In the CO_2_ graph, the *y*-axis is color-coded according to bands commonly used by the American Society of Heating, Refrigerating and Cooling Engineers (ASHRAE): green (< 1000 ppm), yellow (1000–2000 ppm), and red (≥ 2000 ppm). Outliers above 15 *μ*g/m^3^ are not shown in PM_2.5_ and PM_10_ graphs. Note: CO_2_, carbon dioxide; PM_2.5_, fine particulate matter with diameter < 2.5 *μ*m; PM_10_, coarse particulate matter with diameter < 10 *μ*m; PPM, parts per million; F, Fahrenheit.

The mean 7-h CO_2_ concentration during occupied school hours was 1370 ppm (IQR: 1083–1620) for School A classrooms and 1228 ppm (IQR: 788–1460) for School B classrooms. School A’s cafeteria had a mean 7-h CO_2_ concentration of 1273 ppm (IQR: 1064–1450), while School B’s multipurpose room was 723 ppm (IQR: 592–736). Overall, 50% of evaluated rooms (four classrooms and School A’s cafeteria) had mean CO_2_ levels above 1000 ppm, with the remaining two classroom at 981 and 962 ppm (Figure 3a and Table S1).

The 7-h mean levels of both PM_2.5_ and PM_10_ were 4 *μ*g/m^3^ (IQR: 1–7) in School A classrooms, while School B classrooms had lower PM_2.5_ and PM_10_ levels at 2 *μ*g/m^3^ (IQR: 1–3) (Figure 3b). Both schools’ PM_2.5_ and PM_10_ levels were below the 2021 WHO recommended 24-h targets of 15 *μ*g/m^3^ for PM_2.5_ and 45 *μ*g/m^3^ for PM_10_ [29].

During school hours, School A classrooms had a 7-h median temperature of 72.3^°^F (IQR: 71.4–73.2) [22.4°C (IQR:21.9–22.9)] and RH of 37% (IQR: 35–41). School B classrooms had a comparable median temperature of 73°F (IQR: 71.8–74.5) [22.8°C (IQR: 22.1–23.6)] but a lower median RH of 30% (IQR: 28–32) (Figure 3b and Figure S1).

No classrooms exhibited extreme (ICONE index = 5) air stuffiness, which corresponds to CO_2_ levels above 1700 ppm for 5 or more school hours. Four of five classrooms had high (ICONE index = 3) or very high (ICONE index = 4) air stuffiness. We were unable to calculate the ICONE index for one classroom in School B as room occupancy did not meet the normal attendance definition during the study period. Nurses’ offices in both schools showed little to no air stuffiness (ICONE index = 0 or 1) (Table S1 and Table 2).

### 3.5. Viral Detection Results.

In School A and B classrooms, the total air volume sampled was 3675 ft^3^ (104,064 L) for School A and 4172 ft^3^ (118,168 L) for School B over a 9-h period. Air volume sampled in common spaces was 3710 ft^3^ (105,133 L) for School A’s cafeteria and 4261 ft^3^ (120,658 L) for School B’s multipurpose room over a 9-h period (Table S1 and Table 2).

We collected 30 bioaerosol samples: 18 from six classrooms, 6 from common spaces (cafeteria/multipurpose room), and 6 from two nurses’ offices. Of these, 90% (27/30) tested positive for at least one respiratory virus. Among the positive samples, the median number of different viruses detected per sample was three (IQR: 2–3).

Of the 173 nasal swabs collected, 35% (61 samples) from 52 participants tested positive for at least one respiratory virus, with 12% (seven samples) showing codetection of two or more viruses. In classrooms with bioaerosol sampling, 54 swabs were collected from 23 participants. Of these, 43% (23/54 swabs) tested positive for at least one virus: 52% (16/31) at School A and 30% (7/23) at School B (Table S2).

**Figure 4:**
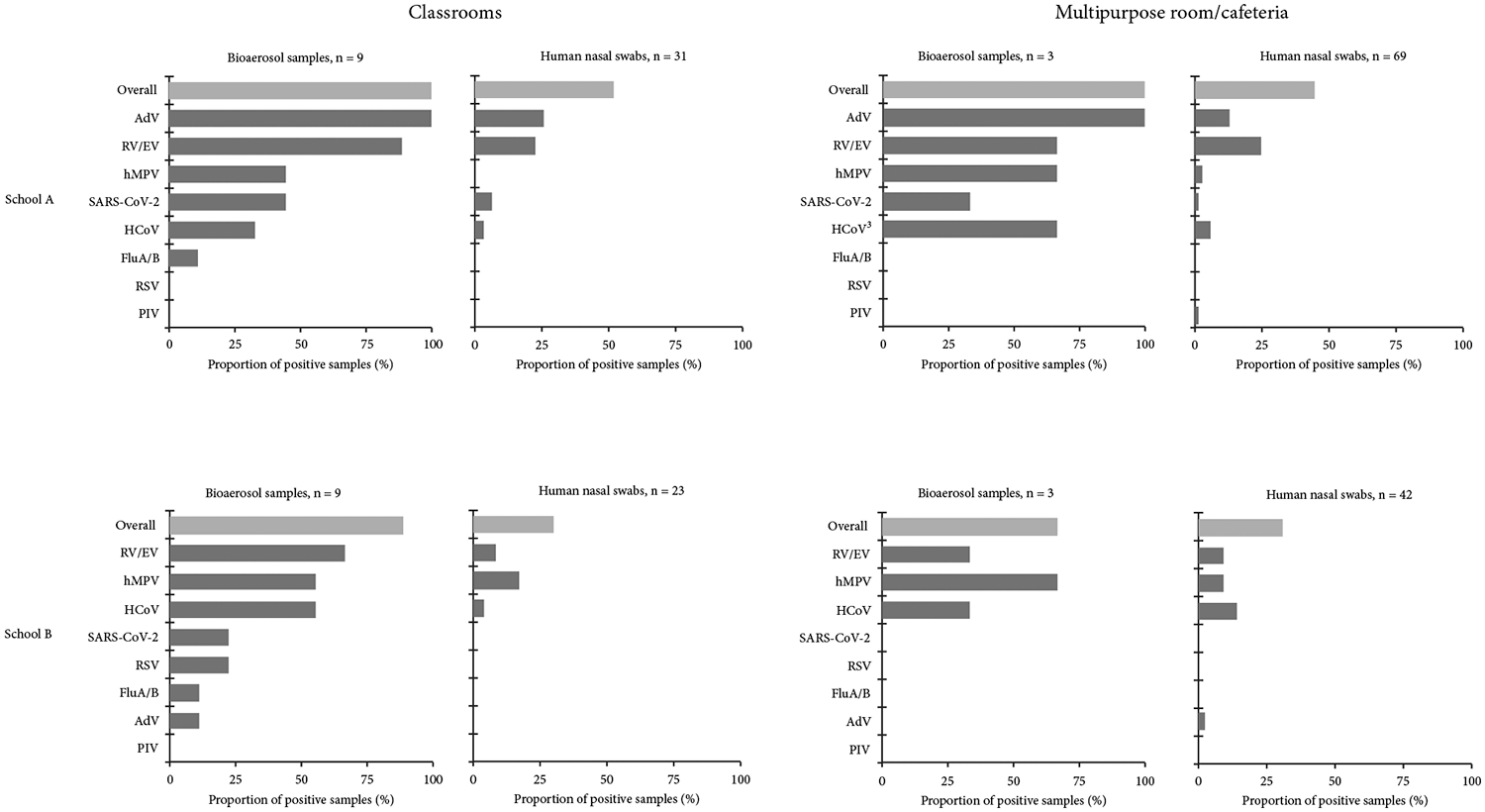
Virus detections by room type in bioaerosol and nasal samples in schools—February–March 2023. This graph shows the proportion of respiratory viruses detected by specific virus and room type in both bioaerosol and nasal swab samples in two elementary schools. Classroom graphs include viral detections from bioaerosol and nasal swab samples limited to student and staff participants in classrooms with bioaerosol sampling. The multipurpose room/cafeteria graphs include nasal swab samples from any student participant, regardless of whether they were also from a classroom with bioaerosol sampling. Human coronavirus types 229E, HKU1, NL63, and OC43 were combined into one category, reported as detection of at least one type. Influenza Types A and B and parainfluenza viruses Types 1–4 were also combined and reported as detection of at least one type. Note: AdV, adenovirus; FluA/B, influenza; hMPV, human metapneumovirus; HCoV, human coronavirus; PIV, parainfluenza virus; RSV, respiratory syncytial virus; RV/EV, rhinovirus/enterovirus.

In School A classrooms, AdV and RV/EV had the highest proportions of detection in both bioaerosol and nasal swab samples. AdV was detected in 100% (9/9) of bioaerosol samples and 26% (8/31) of nasal swabs, while RV/EV was detected in 89% (8/9) of bioaerosol samples and 23% (7/31) of nasal swabs. hMPV (44% [4/9]) and FluA (11% [1/9]) were detected only in bioaerosol samples (Figure 4 and Table S2).

**Figure 5:**
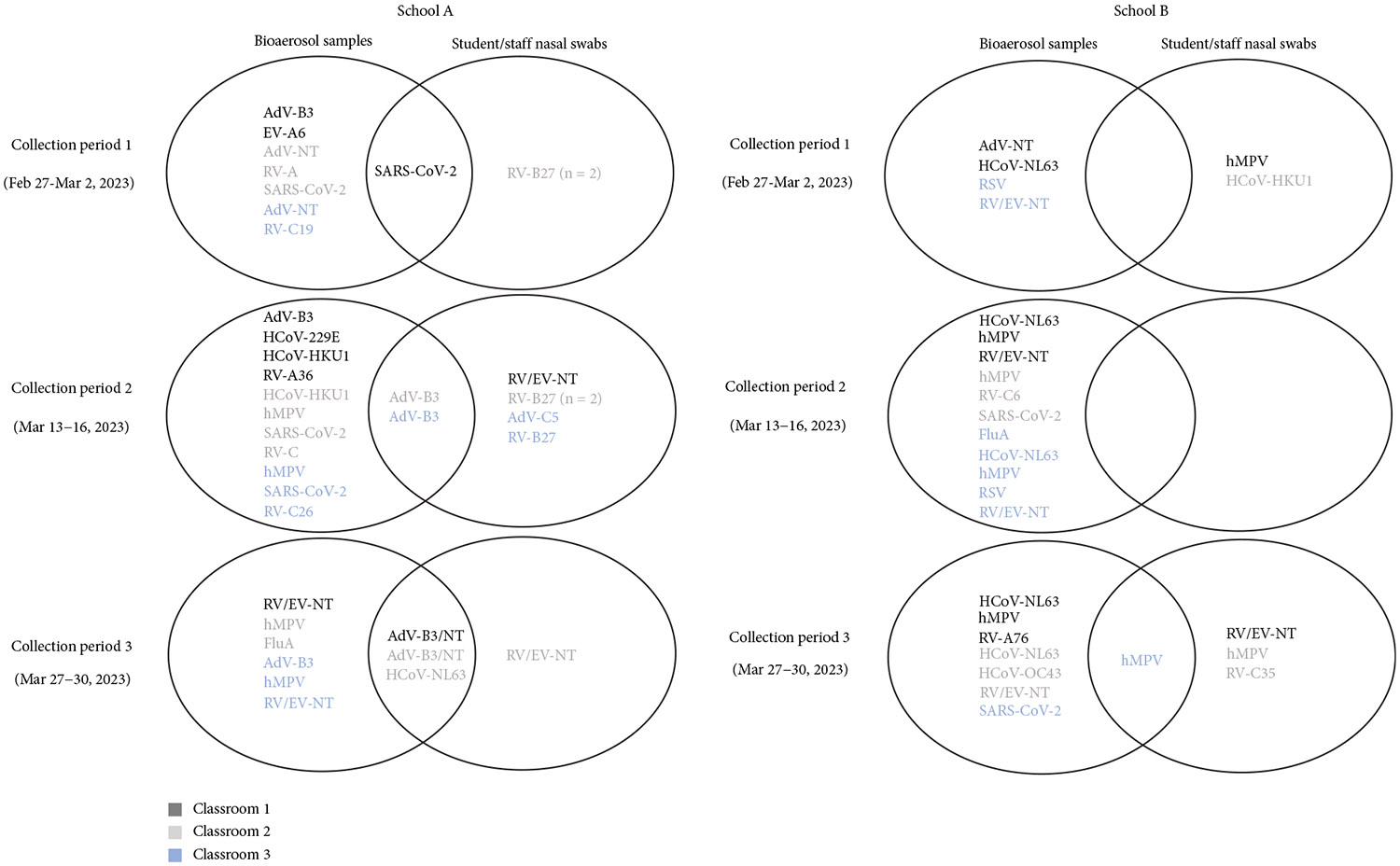
Concordant respiratory virus detections in bioaerosol and nasal samples in school classrooms—February–March 2023. Concordant detections of respiratory viruses in both bioaerosol samples and nasal swabs were assessed in all classrooms from both elementary schools where bioaerosol sampling occurred. Only nasal swabs from participating students/staff in these classrooms were compared. In School A, during collection Period 1, Enterovirus-A6 (a coxsackievirus) was identified by VP4/VP2 sequencing in one bioaerosol sample and is represented as “EV-A6” here. Adenovirus (AdV) or rhinovirus/enterovirus (RV/EV) detections are noted as “NT” (nontypeable) when sequencing was not performed or unsuccessful. During collection Period 3 in School A, AdV-B3 was detected in bioaerosol samples, but nasal swabs were nontypeable. Note: AdV, adenovirus; EV, enterovirus; Flu A, Influenza A; hMPV, human metapneumovirus; HCoV, human coronavirus; NT, nontypeable; RV, rhinovirus; RV/EV, rhinovirus/enterovirus.

Concordant detections of SARS-CoV-2, AdV, and HCoV-NL63 were observed in both bioaerosol and nasal swab samples across all three classrooms in School A, with variations by classroom and collection period (Figure 5). AdV was the most common virus with concordant detection, with AdV Type B3 (AdV-B3) detected in both sample types during Collection Period 2 and in bioaerosol samples during Collection Period 3 (while AdV-positive nasal swabs in Period 3 were nontypeable). RV/EV concordance occurred during Periods 1 and 2, although different types were identified in bioaerosol and nasal swab samples. Of the 7 RV/EV-positive nasal swabs, 5 were sequenced as RV-B27, while 2 were nontypeable (Figure 5).

In School B classrooms, RV/EV, hMPV, and HCoV had the highest proportion detected in both bioaerosol and nasal swab samples. RV/EV was detected in 67% (6/9) of bioaerosol samples and 9% (2/23) of nasal swabs; hMPV in 56% (5/9) of bioaerosol samples and 17% (4/23) of nasal swabs; and HCoV in 56% (5/9) of bioaerosol samples and 4% (1/23) of nasal swabs. RSV (22% [2/9]), FluA (11% [1/9]) and AdV (11% [1/9]) were detected only in bioaerosol samples (Figure 4 and Table S2). Concordant detection of hMPV occurred in one classroom during Collection Period 3, and RV/EV concordance was observed in two classrooms during Collection Period 3, though one sample type was nontypeable (Figure 5).

**Figure 6:**
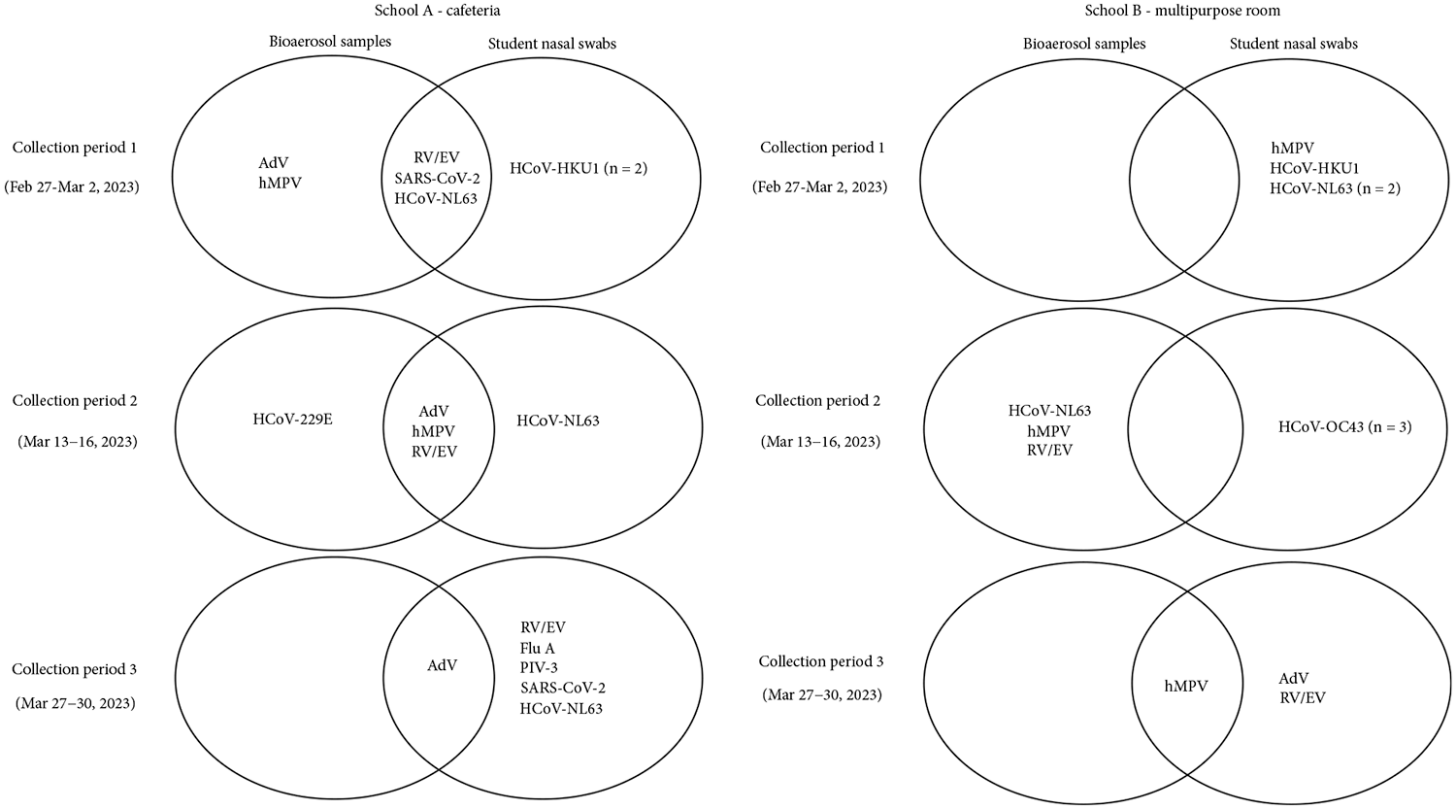
Concordant respiratory virus detections in bioaerosol/nasal samples from school common rooms—February–March 2023. Concordant detections of respiratory viruses in bioaerosol samples and nasal swabs were assessed in the multipurpose room and cafeteria (i.e., common areas) of both elementary schools where bioaerosol sampling occurred. Nasal swabs from all student participants (not limited to those from classrooms with bioaerosol sampling) were compared with bioaerosol samples from these spaces, as teachers do not eat lunch in these areas. Note: AdV, adenovirus; FluA, Influenza A; hMPV, human metapneumovirus; HCoV, human coronavirus; PIV, parainfluenza virus; RV, rhinovirus; RV/EV, rhinovirus/enterovirus.

Assuming all students ate in the cafeteria or multipurpose room (excluding staff who typically eat elsewhere), we collected 111 swabs from 61 students (69 from 40 students in School A; 42 from 21 students in School B) (Table S2). In School A’s cafeteria, AdV and RV/EV were the most frequently detected: AdV in 100% (3/3) of bioaerosol samples and 13% (9/69) of nasal swabs; RV/EV in 67% (2/3) of bioaerosol samples and 25% (17/69) of nasal swabs. In School B’s multipurpose room, hMPV had the highest proportion detected: 67% (2/3) of bioaerosol samples and 10% (4/42) of nasal swabs (Figure 4 and Table S2). Concordant detection of at least one of these respiratory viruses (SARS-CoV-2, HCoV-NL63, RV/EV, AdV, and hMPV) occurred in both bioaerosol and nasal swab samples across all three collection periods (Figure 6).

### 3.6. Frequency of Respiratory Virus Detections by High/Low CO_2_ Concentrations.

Among classrooms with high CO_2_ concentrations (≥ 1000 ppm), viral detection was observed in 48% (20/42) of nasal swabs and 100% (14/14) of bioaerosol samples. In contrast, classrooms with low CO_2_ concentrations (< 1000 ppm) had viral detections in 25% (3/12) of nasal swabs and 75% (3/4) bioaerosol samples. Results suggest a trend toward higher viral detections in classrooms with high CO_2_ concentrations; however, no statistically significant associations were found with viral detections in either nasal swabs (chi-square, *p* = 016) or bioaerosol samples (Fisher’s exact, *p* = 022) (Table 3).

## 4. Discussion

We examined the link between respiratory virus detection and IAQ in elementary school classrooms and nonclassroom settings using bioaerosol sampling and nasal swabs from students and staff. Most bioaerosol samples (90%) and about one-third (35%) of nasal swabs detected at least one respiratory virus. In classrooms with bioaerosol sampling, almost half (43%) of nasal swabs tested positive, with higher detection in School A classrooms (52%). Four classrooms showed concordant virus detection in both bioaerosol and nasal swab samples. AdV-B3 was the predominant type in AdV sequenced samples, while RV/EV types varied or were non-typeable, with RV-B27 predominant in nasal swabs and varied RV/EV types in bioaerosol samples. Our findings show that median CO_2_ concentrations often exceeded 1000 ppm in classrooms, suggesting inadequate ventilation, as ASHRAE notes that higher CO_2_ concentrations are indicative of lower OA ventilation rates and may contribute to increased airborne transmission risk [30]. Two-thirds of classrooms had median CO_2_ levels above 1000 ppm during school hours, and the ICONE across all classrooms was three, indicating high air stuffiness. Notably, classrooms with CO_2_levels ≥ 1000 ppm showed a higher proportion of viral detections in bioaerosol and nasal swab samples, highlighting the potential role of IAQ in respiratory virus transmission. While this pattern may suggest a possible relationship, the lack of statistical significance indicates that a larger sample size may be needed to draw more definitive conclusions.

Our findings in elementary schools align with a review article [31], showing ventilation rates often fall below current CDC and ANSI/ASHRAE recommendations. None of the evaluated spaces met the CDC recommended 5 ACH 5, and only one exceeded the ANSI/ASHRAE 62.1-2002 standard for OA supply [24, 32]. Several studies have reported similarly low ventilation rates in schools—typically below 10 L/s per person, equivalent to ~4 ACH [2, 10, 11, 31, 33]—and a recent report recommends even higher rates (e.g., > 6 ACH or > 14 L/s per person) to reduce airborne respiratory disease transmission [2]. Additionally, the new ASHRAE Standard 241 recommends 20 L/s per person for infection risk management to achieve higher levels of infection risk mitigation [34, 35]. Poor ventilation has also been linked to worse student health, attendance, and performance [10, 11, 31]. Elevated CO_2_ levels, a proxy for inadequate ventilation, have been associated with increased SARS-CoV-2 cases in schools and lower respiratory tract infections in households [36, 37]. In one study, classrooms with *CO*_2_ > 1000 *ppm* had higher SARS-CoV-2 incidence, while those with MERV13 filters had lower incidence compared to MERV11. A study of IAQ and lower respiratory tract infections in Canadian households found a significant association between mean indoor CO_2_ levels and lower respiratory tract infection. Another study found that ventilation rates > 10 L/s per student reduced SARS-CoV-2 infection risk by 80% in mechanically ventilated classrooms [38]. Collectively, these findings support our conclusion that poor ventilation—including elevated CO_2_ levels—may increase the risk of respiratory virus exposure and transmission in schools.

Improving IAQ through increased ventilation and filtration may reduce respiratory virus transmission risk yet may raise energy and HVAC costs. The US Department of Energy estimates that energy costs are the second-largest expense for K-12 school districts after salaries [39]. However, a review article found that the per-person incremental cost of higher capacity HVAC systems in Florida schools was small—approximately $2–$3 per person per year—and likely even lower in milder climates [31]. A 2020 US Government Accountability Office survey found that 41% of school districts need to update or replace HVAC in at least half of their schools, affecting nearly 36,000 US schools [40]. More support tools are needed to help school administrators implement complex technical recommendations beyond their areas of expertise. Ventilation and filtration improvements should balance energy use and budget constraints with the goal of reducing respiratory virus transmission and other adverse health effects associated with poor IAQ. Importantly, improvements in school IAQ could also reduce respiratory illness, student absenteeism, and caregiver work loss—resulting in societal and economic benefits.

Our study is one of the few to conduct bioaerosol sampling of respiratory viruses alongside human specimen collection in indoor spaces, particularly schools. Only two previous studies have investigated this in a school environment [9, 41]. We found simultaneous detection of several respiratory viruses in both bioaerosol and nasal swab samples across classrooms and common spaces, with higher detection of respiratory viruses in bioaerosol samples compared to a study by Banholzer et al. Key differences in the Banholzer study include the use of PACs with HEPA filters and its focus on secondary schools (students aged 14–17 years), while our study involved primary schools (students with a median age of 8 years) without PACs. Young children are more likely to have higher prevalence of respiratory virus infections, and HEPA filters are known to be highly effective at removing infectious particles [7]. Additionally, bioaerosol sampling collection time was not reported, which could affect viral detection rates. Our results demonstrate that bioaerosol sampling can identify circulating respiratory viruses, aligning with previous studies that successfully detected multiple respiratory viruses via bioaerosol sampling in educational settings [8, 16]. IAQ assessments supplemented by bioaerosol sampling can aid epidemiologic investigations. These tools can further complement traditional methods that may come with limitations such as human specimen collection, which can be costly and is dependent on participation.

This analysis is subject to several limitations. First, human samples were only collected from participants opting into surveillance, representing a small proportion of the classroom population. Viruses detected in bioaerosol samples, but not nasal swabs, could have originated from individuals who did not submit swabs. Second, there was a 48h delay between nasal swab and bioaerosol sampling, which could result in missed overlap in viral shedding, given the short duration of some respiratory infections. Third, we did not attempt viral culture for positive samples to confirm viral viability, so detection should not be assumed to indicate viable virus, although it may suggest recent replication in individuals occupying the space where detection occurred. Culturing virus from bioaerosol samples in particular is unlikely, as virus viability is degraded by the mechanical stress of impaction and dehydration during sampling. However, our findings should be interpreted as indicators of potential exposure, not definitive evidence of transmission risk. Fourth, partial genome Sanger sequencing limited our ability to sequence some samples and draw epidemiologic conclusions. Fifth, data collection occurred in February–March 2023, potentially affecting virus detection due to seasonality. Sixth, no field blanks were collected to test for RNA contamination in bioaerosol samplers, though one sampler was tested before use and showed no contamination. Seventh, HVAC run times were measured on only 1 day per collection period and assumed to reflect typical conditions, which may affect ventilation results. Finally, this was a small pilot conducted in two schools, and larger studies are needed to confirm observed trends.

## 5. Conclusions

Limited research has linked respiratory virus detection in bioaerosol and nasal swab samples in congregate settings. This pilot study collected human health (nasal swabs and demographics) and environmental data (IAQ measures, ventilation rates, and bioaerosol sampling) from two elementary schools. Ventilation rates varied, with some areas showing inadequate ventilation, indicated by elevated CO_2_ levels and total clean ACH values below recommended levels. Higher viral detections in bioaerosol samples from classrooms with elevated CO_2_ concentrations suggest a potential association between IAQ, ventilation, and viral transmission. However, the findings were not statistically significant, and more data is needed to confirm this association. Bioaerosol sampling paired with nasal swab collection revealed the presence of viruses in both sample types, indicating potential airborne transmission in these environments. These findings support the utility of combining IAQ assessments with bioaerosol and respiratory sample collection for epidemiologic and surveillance purposes. Integrating ventilation assessments with respiratory virus surveillance offers valuable insights into the relationship between IAQ and infectious disease transmission in schools, helping to better understand the impact of prevention strategies, including optimizing ventilation and other environmental control strategies. Larger studies are needed to confirm the association between respiratory virus detection through bioaerosol sampling, ventilation rates, and IAQ conditions and to guide effective environmental control strategies in schools.

## Supplementary Material

Supplementary Results

Additional supporting information can be found online in the Supporting Information section. *(Supporting Information)* Supporting tables provide data on building, ventilation, and room characteristics as well as virus detections in nasal swabs and bioaerosol samples. Table S1: Building, ventilation, room characteristics, and indoor air quality indicators during school hours in two elementary schools—Kansas City, Missouri, February–March 2023. Table S2: Respiratory virus detections in bioaerosol samples and student/staff nasal swabs collected in two elementary schools—Kansas City, Missouri, February–March 2023.

## Figures and Tables

**TABLE 1:** Characteristics of elementary school students/staff participating in school-based respiratory virus testing, February–March 2023, Missouri.

	Total (*N* = 100)	School A (*N* = 62) *N* (col %)	School B (*N* = 38)
Student participants	61 (61.0)	40 (64.5)	21 (55.3)
Students in bioaerosol sampled classrooms^a^	17 (27.9)	10 (25.0)	7 (33.3)
Median age in years (IQR)	8.4 (6.8, 10.2)	8.2 (6.6, 10.1)	9.1 (6.9, 10.7)
Staff participants	39 (39.0)	22 (35.5)	17 (44.7)
Staff in bioaerosol sampled classrooms	8 (20.5)	4 (18.2)	4 (23.5)
Median age in years (IQR)	39.6 (31.5, 53.5)	40.1 (33, 55)	38.8 (31.5, 51.4)
Gender^b^			
Female	68 (68.0)	40 (64.5)	28 (73.7)
Male	28 (28.0)	21 (33.9)	7 (18.4)
Race/ethnicity^c^			
White, NH	60 (60.0)	34 (54.8)	26 (68.4)
Hispanic/Latino	15 (15.0)	9 (14.5)	6 (15.8)
Some other race, NH^d^	16 (16.0)	13 (21.0)	3 (7.9)
Number of household members^e^			
1–4	61 (61.0)	33 (53.2)	28 (73.7)
5 or more	34 (34.0)	26 (41.9)	8 (21.1)
Enrolled in national school lunch program^f^	25/61 (41.0)	20/40 (50.0)	5/21 (23.8)
Vaccination status			
Influenza vaccine (current season)	36 (36.0)	20 (32.3)	16 (42.1)
Any COVID-19 vaccine	67 (67.0)	39 (62.9)	28 (73.7)
Bivalent COVID-19 vaccine	16 (16.0)	6 (9.7)	10 (26.3)
Total nasal swabs collected during pilot period^g^	173	101	72
Surveillance swabs	166 (96.0)	97 (96.0)	69 (95.8)
'On demand’ symptomatic swabs	7 (4.0)	4 (4.0)	3 (4.2)

Abbreviations: IQR, interquartile range; NH, non-Hispanic/Latino.

a There were a total of six classrooms with bioaerosol sampling, three classrooms per school.

b There were four participants with unknown gender (*n* = 1, School A; *n* = 3, School B).

c There were nine participants with unknown race and/or ethnicity (*n* = 6, School A; *n* = 3, School B).

d The “some other race, NH” group includes black, NH; Asian, NH; native Hawaiian or Pacific Islander, NH; and multiracial, NH.

e The number of household members was unknown for five participants (*n* =3, school A; *n* =2, School B).

f The official name of the national free or reduced-price lunch program. These data were collected for student participants only. Four students from school B had unknown enrollment in the National School Lunch Program.

g Analysis was limited to swabs collected during February 27–March 2, March 13–16, and March 27–30, 2023.

**TABLE 2:** Building, ventilation characteristics, and room occupancy in two elementary schools, Kansas City, Missouri, 2022–2023.

Year built	All classrooms	School A 1955 Nurses’ office 1	Cafeteria	All classrooms	School B 1959 Nurses’ office 2	Multipurpose room
Room occupancy						
Expected occupancy	25	5	240	25	3	150
Observed average occupancy (range)^a^	22 (18–40)	4 (3–5)	78 (65–105)	19 (16–30)	3 (3–4)	93 (80–105)
Percentage of time at 50% occupancy^b^	71%	76%	50%	61%	73%	60%
Median participants/classroom (range)	4 (2–4)	NA	NA	2 (2–4)	NA	NA
Participants in classrooms with bioaerosol sampling/maximum occupants (%)	18%	NA	NA	15%	NA	NA
Room specifications						
Room area (ft^2^)	811	263	2926	768	135	2418
Room volume (ft^3^)	7007	2365	43,228	6424	1078	36,836
Bioaerosol sampling						
Total sampling time (hours)^c^	27	27	27	30	30	30
Mean (ft^3^) air volume sampled^d^	3671	3692	3706	4167	4214	4257
Ventilation conditions^e^ (%)						
Door open	74%	2%	100%	59%	98%	51%
HVAC system run-time (using MERV8 filtration)^f^	24%	33%	76%	5%	18%	47%
Windows open	0%	NA	0%	0%	NA	0%
Supply and outdoor air rates						
Total supply air rate, measured (cfm, [L/s])	894 [422]	1213 [572]	10,446 [4930]	7497 [3538]	668 [315]	10,448 [4931]
Normalized supply air rate (cfm, [L/s])^g^	215 [101]	400 [189]	7939 [3747]	373 [176]	120 [57]	4911 [2318]
Outdoor air rate, recommended (cfm, [L/s])^h^	321 [151]	87 [41]	761 [359]	295 [139]	54 [25]	843 [398]
Outdoor air rate, estimated (cfm, [L/s])^i^	21 [10]	40 [19]	794 [375]	37 [17]	12 [6]	491 [232]
Room meets code for outdoor air rate?^j^	No	No	Yes	No	No	No
Air stuffiness index (ICONE score)^k^	3	1	—^l^	3	0	—

Abbreviations: CFM, cubic feet/minute; HVAC, heating, ventilation, air conditioning; ICONE, indice confinement d’air dans les ecoles; L/s, liters/second; NA, not applicable.

a The maximum number of occupants per collection period was recorded. The reported value is the average of the maximum number of occupants across all three collection periods, with the range representing the maximum occupancy per period.

b This represents the proportion of time the room was occupied at 50% of the observed average occupancy during school hours (for classrooms/nurses’ offices) or lunch hours (estimated between 11:00 a.m. and 1:00 p.m. for cafeteria/multipurpose room).

c Total sampling time was calculated as total minutes sampled × 3 collection periods = total sampling time in hours.

d This is the mean air volume sampled in cubic feet across all collection periods per room by school. Air sampled per room is calculated as follows: [*total sampling time in minutes* × *sampling rate* of 7.06 *cfm* (*equivalent* to 200 *L*/min)] in ft^3^ . For example, (520 *minutes* × 7.06 *cfm*) = 3671 ft^3^ air sampled.

e Ventilation conditions (doors/windows open or closed, HVAC system on or off) were recorded every 30-min during school hours on the IAQ data collection day of each collection period. Reported proportions are averages across all 3 days, despite variability at each timepoint.

f HVAC run-time estimated from observing whether system on/off every 30-min during 1 day per collection period. The thermostat for one classroom in School B is linked to an adjacent classroom’s thermostat, which is only occupied before or after school hours. Since the thermostat’s classroom is unoccupied during the school day, the HVAC system was not activated.

g Normalized supply air rate for each evaluated space using estimated HVAC run-time during school hours: *normalized supply air rate = total supply air rate* × *estimated* HVAC *run – time* = 823 *cfm* × 0.26 = 214 *cfm*.

h Calculated recommended outdoor air rate per ANSI/ASHRAE Standard 62.1-2022 using the following occupancy categories: classrooms: ages 5–8; classrooms: ages 9 plus; daycare sickroom (for nurses’ office); and multiuse assembly (for cafeteria/multipurpose room). Values calculated as follows: *outdoor air* (OA) *rate, recommended* = (*rate of outdoor air per person x observed number of occupants*) + (*outdoor air rate for the area* × *room area*). For example, OA rate, *rec* = (10 c*fm/person* × 19 *people*) + (0.12 *cfm*/ft^2^ × 811 ft^2^) = 287 *cfm* for a classroom with children ages 5–8 years.

i Estimated OA intake into the space by multiplying normalized supply air rate by 10% (assuming open setting for OA dampers allowed this amount of intake). For example, OA, *estimated* = 214 *cfm* ×0.10 = 21 *cfm* for a classroom.

j If estimated OA rate exceeds the recommended OA rate, the space meets current code (per ANSI/ASHRAE Standard 62.1-2022).

k An index assessing indoor air “stuffiness” during periods with at least 50% occupancy for 5+ h. It converts average CO_2_ concentrations into a score of 0–5, where 0 = no stuffiness (*CO*_2_ ≤ 1,000 *ppm* 100% of the time) and 5 = extreme stuffiness (CO_2_ > 1,700 ppm 100% of the time). One classroom was excluded as it did not meet the occupancy requirement. For the complete formula, refer to the Materials and Methods section.

l ICONE score was not calculated for the cafeteria or multipurpose room as these spaces did not meet the criteria of 50% occupancy for a minimum of 5 h.

**TABLE 3:** Respiratory viral detection by sample type, CO_2_ levels in elementary school classrooms—February–March 2023.

CO_2_ concentration, 7 h mean^a^	Viral detections–nasal swabs^b^				Viral detections–bioaerosol samples			
	Detected	Not detected	Total		Detected	Not detected	Total	
	*N* (row %)		*N* (%)	*p* value	*N* (row %)		*N* (%)	*p* value
High CO_2_ (≥ 1000 ppm)	20 (47.6%)	22 (52.4%)	42 (77.8%)	0.16	14 (100.0%)	0	14 (77.8%)	0.22
Low CO_2_ (< 1000 ppm)	3 (25.0%)	9 (75.0%)	12 (22.2%)		3 (75.0%)	1 (25.0%)	4 (22.2%)	
Total	23 (42.6%)	31 (57.4%)	54 (100%)		17 (94.4%)	1 (5.6%)	18 (100.0)	

Abbreviation: PPM, parts per million.

a CO_2_ data were collected using the TSI AirAssure 8144-4 monitor on bioaerosol sampling days. Respiratory nasal swabs were collected 2 days earlier. CO_2_ concentrations were classified as high (≥ 1000 ppm) or low (< 1000 ppm) based on 7-h mean values for each collection period and classroom.

b Nasal swabs collected from participants in classrooms with bioaerosol sampling.

## Data Availability

The data that support the findings of this study are available on request from the corresponding author. The data are not publicly available due to privacy or ethical restrictions.
