# Supplementary material for: Indoor Air Quality Conditions and Respiratory Virus Detections in Elementary Schools—Kansas City, Missouri, February–March 2023
Source: Indoor Air. Author manuscript; Available in PMC 2025 Sep 9. (PMC12416244; doi:10.1155/ina/9935344)

**Supplemental Results**

**Supplemental Table 1. Building, ventilation, room characteristics and indoor air quality indicators during school hours in two elementary schools—Kansas City, Missouri, February–March, 2023**

|  | **School A** | | | | | **School B** | | | | |
| --- | --- | --- | --- | --- | --- | --- | --- | --- | --- | --- |
|  | **Classroom 1** | **Classroom 2** | **Classroom 3** | **Nurse's office 1** | **Cafeteria** | **Classroom 1** | **Classroom 2** | **Classroom 3** | **Nurse's office 2** | **Multipurpose Room** |
| **Room Occupancy** |  |  |  |  |  |  |  |  |  |  |
| Expected occupancy | 25 | 25 | 25 | 5 | 240 | 25 | 25 | 25 | 3 | 150 |
| Observed average  occupancy, (range)*^a^* | 19  (18–20) | 28  (22–40)*^b^* | 20  (18–24) | 4  (3–5) | 78  (65–105) | 19  (16–22) | 19  (14–30)*^c^* | 18  (17–19) | 3  (3–4) | 93  (80–105) |
| Percentage of time at 50%  occupancy*^d^* | 76% | 67% | 71% | 76% | 50% | 73% | 42% | 67% | 73% | 60% |
| Median participants/  classroom, (range) | 3 (3–4) | 4 (3–4) | 4 (2–4) | NA | NA | 2 (2–2) | 4 (3–4) | 2 (2–3) | NA | NA |
| Participants in bioaerosol  sampled classrooms/ Max  occupants (%) | 20% | 17% | 17% | NA | NA | 9% | 21% | 16% | NA | NA |
| **Room Specifications** |  |  |  |  |  |  |  |  |  |  |
| Room area (ft^2^) | 811 | 816 | 806 | 263 | 2926 | 790 | 789 | 726 | 135 | 2418 |
| Room volume (ft^3^) | 7132 | 6977 | 6913 | 2365 | 43228 | 6636 | 6584 | 6052 | 1078 | 36836 |
| **Bioaerosol Sampling** |  |  |  |  |  |  |  |  |  |  |
| Total sampling time, hours*^e^* | 27 | 27 | 27 | 27 | 27 | 30 | 30 | 30 | 30 | 30 |
| Mean air volume sampled (ft^3^)*^f^* | 3671 | 3664 | 3678 | 3692 | 3706 | 4144 | 4165 | 4193 | 4214 | 4257 |
| **Ventilation Conditions*^g^*, %** |  |  |  |  |  |  |  |  |  |  |
| Door open | 86% | 81% | 55% | 2% | 100% | 22% | 56% | 100% | 98% | 51% |
| HVAC system running | 24% | 24% | 24% | 33% | 76% | 4% | 11% | 0% *^h^* | 18% | 47% |
| Windows open | 0% | 0% | 0% | NA | 0% | 0% | NA | 0% | NA | 0% |
| **Supply and Outdoor Air Rates** |  |  |  |  |  |  |  |  |  |  |
| Total Supply Air Rate, Measured (cfm, [L/s]) | 823 [389] | 778 [367] | 1082 [510] | 1213 [572] | 10446 [4930] | 8695 [4102] | 6999 [3310] | 6798 [3205] | 668 [315] | 10448 [4931] |
| Normalized Supply Air Rate (cfm [L/s])*^i^* | 198 [93] | 187 [88] | 260 [122] | 400 [189] | 7939 [3747] | 348 [164] | 770 [363] | 0 | 120 [57] | 4911 [2318] |
| Outdoor Air Rate, Recommended (cfm [L/s])*^j^* | 287 [135] | 378 [178] | 297 [140] | 87 [41] | 761 [359] | 332 [157] | 258 [134] | 267 [126] | 54 [25] | 843 [398] |
| Outdoor Air Rate, Estimated (cfm [L/s])*^k^* | 20 [9] | 19 [9] | 26 [12] | 40 [19] | 794 [375] | 35 [16] | 77 [36] | 0 | 12 [6] | 491 [232] |
| Room meets code for Outdoor Air Rate?*^l^* | No | No | No | No | Yes | No | No | No | No | No |
| **Air Stuffiness Index (ICONE Score)*^m^*** | 3 | 4 | 3 | 1 | -- | 4 | -- | 2 | 0 | -- |
| **IAQ Indicators*^n^*, 7-hr median (IQR)** |  |  |  |  |  |  |  |  |  |  |
| CO_2_ concentration (ppm) | 1271  (1083–1502) | 1439  (1101–1670) | 1427 (1054–1644) | 923 (689–1037) | 1204 (1064–1450) | 1663 (1176–2236) | 923 (738–1137) | 1014 (750–1183) | 734 (661–793) | 661 (592–736) |
| PM_2.5_ (µg/m^3^) | 6 (1–7) | 4 (1-6) | 6 (1–7) | 5 (2–7) | 6 (2–7) | 2 (1–2) | 2 (1–3) | 3 (1–4) | 2 (1–3) | 3 (1–4) |
| PM_10_ (µg/m^3^) | 6 (1–7) | 4 (1–6) | 6 (1–7) | 5 (2-7) | 6 (2–7) | 2 (1–2) | 2 (1–3) | 3 (1–4) | 2 (1–3) | 3 (1–4) |
| Temperature (°F) | 72.0 (71.2–72.7) | 72.7 (70.9–73.6) | 72.9 (71.6–73.6) | 72.3 (72.0–72.9) | 75.2 (73.2–76.1) | 73.4 (72.7–74.1) | 71.2 (71.1–72.0) | 75.9 (73.9–77.4) | 76.1 (75.7–76.6) | 70.3 (69.4–71.2) |
| \| Temperature (°C) \| 22.2 (21.8–22.6) \| 22.3 (21.6–23.1) \| 22.6 (22.0–23.1) \| 22.4 (22.2–22.7) \| 23.8 (22.9–24.5) \| 22.9 (22.6–23.4) \| 21.9 (21.7–22.2) \| 24.3 (23.3–25.2) \| 24.6 (24.3–24.8) \| 21.3 (20.8–21.8) \| \| --- \| --- \| --- \| --- \| --- \| --- \| --- \| --- \| --- \| --- \| --- \| | 22.2 (21.8–22.6) | 22.6 (21.6–23.1) | 22.7 (22.0–23.1) | 22.4 (22.2–22.7) | 24.0 (22.9–24.5) | 23.0 (22.6–23.4) | 21.8 (21.7–22.2) | 24.4 (23.3–25.2) | 24.5 (24.3–24.8) | 21.3 (20.8–21.8) |
| Relative humidity (%) | 38 (36–42) | 37 (35–42) | 37 (34–40) | 35 (30–39) | 40 (35–45) | 32 (29–34) | 31 (28–32) | 28 (27–30) | 25 (23–26) | 30 (28–31) |

NA=Not Applicable; HVAC=Heating, Ventilation, Air Conditioning; CFM=Cubic Feet per Minute; L/s=Liters per second; ACH= Air Changes per Hour; F=Fahrenheit; ICONE= Indice de CONfinement d'air dans les Ecoles; IAQ=Indoor Air Quality; IQR=Interquartile Range; PPM=Parts per million; PM=particulate matter

*^a^* This is the average maximum occupancy across all three collection periods, with the range representing the maximum occupancy per collection period.

*^b^* Another class joined for ~30 minutes during school hours bringing occupancy to 40 students; otherwise, 24 students were present.

*^c^* Two classes shared the classroom for 1 hour with a maximum occupancy of 30, otherwise, 14 students occupied the classroom.

*^d^* Proportion of time the room was at ≥50% of its average observed occupancy during school hours (classrooms/nurses’ offices) or lunch hours (11:00am-1:00pm for cafeteria/multipurpose room).

*^e^* Total sampling time was calculated by multiplying total sample time in minutes by the sample volume rate (7.06 cfm [200 L/min]), then converting sampling time to hours.

*^f^* Mean air volume sampled (cubic feet) across all collection periods: (total sampling time, minutes x sampling rate of 7.06 cfm), e.g., (520 minutes x 7.06 cfm)=3671 ft^3^

*^g^* Ventilation conditions, including door/window open or closed, and HVAC system on or off, were recorded one day per collection period during occupied hours, with averages representing the average across all 3 measurement days, although there was variability across the three measured timepoints.

*^h^* This classroom’s thermostat was linked to an adjacent room used only outside school hours, preventing HVAC system activation based on temperature or humidity setpoints.

*^i^* The supply air rate during school hours was normalized using the estimated HVAC run-time: total supply air rate x estimated HVAC run time = 823 cfm x 0.24= 198 cfm.

*^j^* The recommended outdoor air rate (OA rec), per ANSI/ASHRAE 62.1-2022, was estimated based on space type, occupancy and area: OA rec = (OA rate per person x total occupancy) + (area OA rate x area in ft^2^). For example, for a classroom with children aged 5-8 years and 811 ft^2^: (10 cfm/person)(19 people)+ (0.12 cfm/ft^2^)(811 ft^2^) = 287 cfm.

*^k^* The OA delivered was estimated as: OA Estimated = normalized total supply air x 0.10 (estimated 10% outdoor air), e.g., OA estimated= 198 cfm x 0.10 = 20 cfm.

*^l^* If the estimated OA rate exceeds the recommended OA rate the space meets current ANSI/ASHRAE 62.1-2022 standards.

*^m^* Developed as an index to assess indoor air 'stuffiness' during occupied periods with normal attendance at 50% for a minimum of 5 hours based on average CO_2_ concentrations, scored 0-5, with 0=no stuffiness (CO_2_ ≤1,000 ppm 100% of the time) and 5= extreme stuffiness (CO_2_ >1,700 ppm 100% of the time). For complete formula, see methods section.

*^n^* Indoor Air Quality indicators were measured using TSI AirAssure™ 8144-6 monitor during occupied school hours and averaged across all collection periods for each space.

**Supplemental Table 2. Respiratory virus detections in bioaerosol samples and student/staff nasal swabs collected in two elementary schools—Kansas City, Missouri, February–March, 2023**

|  |  | **School A** | | | **School B** | | |  | **School A** | | | **School B** | |
| --- | --- | --- | --- | --- | --- | --- | --- | --- | --- | --- | --- | --- | --- |
|  | **Total** | **Classrooms with air sampling*^a^*** | **Cafeteria*^b^*** | **Nurse’s**  **Office 1** | **Classrooms with air sampling*^a^*** | **Multipurpose room*^b^*** | **Nurse’s**  **Office** |  | **Total*^c^*** | **Classrooms with air sampling*^a^*** | **Cafeteria*^b^*** | **Classrooms with air sampling*^a^*** | **Multipurpose room*^b^*** |
|  | **N**  **(Col %)** | **N**  **(Col %)** | **N (Col %)** | **N**  **(Col %)** | **N**  **(Col %)** | **N**  **(Col %)** | **N**  **(Col %)** |  | **N**  **(Col %)** | **N**  **(Col %)** | **N**  **(Col %)** | **N**  **(Col %)** | **N**  **(Col %)** |
| **Bioaerosol sampling** |  |  |  |  |  |  |  | **Human Nasal Swabs** |  |  |  |  |  |
| Number samples collected | 24 | 9 | 3 | 3 | 9 | 3 | 3 | Total participating students/staff | 100 | 13 | 40 | 10^a^ | 21 |
| ≥1 respiratory virus detected | 22 (91.7) | 9 (100) | 3 (100) | 3 (100) | 8 (88.9) | 2 (66.7) | 2 (66.7) | Swabs collected | 173 | 31 | 69 | 23 | 42 |
| ≥2 respiratory viruses detected | 20/22 (90.9) | 9/9  (100) | 2/3  (66.7) | 3/3  (100) | 8/8  (100) | 1/2  (50) | 1/2  (50) | ≥1 respiratory virus detected | 61  (35.3) | 16  (51.6) | 31  (44.9) | 7  (30.4) | 13  (31.0) |
|  |  |  |  |  |  |  |  | ≥2 respiratory viruses detected | 7/61 (11.5) | 2/16  (12.5) | 3/31  (9.7) | 0 | 2/13  (15.4) |
| **Detections by virus** |  |  |  |  |  |  |  | **Detections by virus** |  |  |  |  |  |
| Adenovirus | 13 (54.2) | 9 (100) | 3 (100) | 2 (66.7) | 1 (11.1) | 0 | 1 (33.3) | Adenovirus | 10 (5.8) | 8 (25.8) | 9 (13.0) | 0 | 1 (2.4) |
| hMPV | 13 (54.2) | 4 (44.4) | 2 (66.7) | 2 (66.7) | 5 (55.6) | 2 (66.7) | 1 (33.3) | hMPV | 10 (5.8) | 0 | 2 (2.9) | 4 (17.4) | 4 (9.5) |
| RV/EV | 17 (70.8) | 8 (88.9) | 2 (66.7) | 1 (33.3) | 6 (66.7) | 1 (33.3) | 0 | RV/EV | 26 (15.0) | 7 (22.6) | 17 (24.6) | 2 (8.7) | 4 (9.5) |
| Flu A/B*^d^* | 2 (8.3) | 1 (11.1) | 0 | 0 | 1 (11.1) | 0 | 0 | Flu A/B*^d^* | 2 (1.2) | 0 | 0 | 0 | 0 |
| RSV | 2 (8.3) | 0 | 0 | 0 | 2 (22.2) | 0 | 0 | RSV | 0 | 0 | 0 | 0 | 0 |
| Pflu1-4*^e^* | 0 | 0 | 0 | 1 (33.3) | 0 | 0 | 0 | Pflu1-4*^e^* | 2 (1.2) | 0 | 1 (1.5) | 0 | 0 |
| SARS-CoV-2 | 7 (29.2) | 4 (44.4) | 1 (33.3) | 2 (66.7) | 2 (22.2) | 0 | 1 (33.3) | SARS-CoV-2 | 5 (2.9) | 2 (6.5) | 1 (1.5) | 0 | 0 |
| HCoVs*^f^* | 11 (45.8) | 3 (33.3) | 2 (66.7) | 0 | 5 (55.6) | 1 (33.3) | 1 (33.3) | HCoVs*^f^* | 13 (7.5) | 1 (3.2) | 4 (5.8) | 1 (4.4) | 6 (14.3) |

AdV=adenovirus; FluA=influenza A; FluB=influenza B; hMPV=human metapneumovirus; HCoVs=human coronaviruses; PIV=parainfluenza virus; RSV=respiratory syncytial virus; RV/EV=rhinovirus/enterovirus.

*^a^* Only includes students and staff from classrooms where bioaerosol sampling was performed (i.e., excludes nurses’ offices and common rooms).

*^b^* Includes all student participants.

*^c^* Includes all participating students and staff. Three samples were collected among staff from the nurses’ offices in both schools with a detection of RV/EV in one sample.

*^d^*Influenza A and B were combined into one category and reported as detection of at least 1 type.

*^e^* Parainfluenza viruses type 1-4 were combined into one category and reported as detection of at least 1 type.

*^f^* Human coronavirus types 229E, HKU1, NL63, OC43 were combined into one category and reported as detection of at least 1 type.

**Supplemental Figure 1. Indoor Air Quality (IAQ) variability by Temperature in Celsius and room type in two elementary schools—Kansas City, Missouri, February**–**March 2023.**


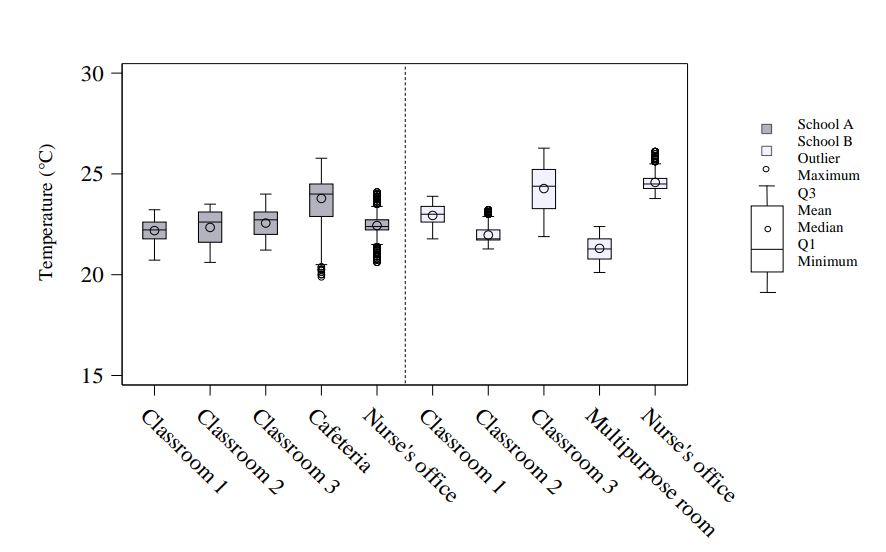

Supplement: Supplementary Results [file NIHMS2106477-supplement-Supplementary_Results.docx]
